# Meta-analysis Reveals the Prognostic Value of Circulating Tumour Cells Detected in the Peripheral Blood in Patients with Non-Metastatic Colorectal Cancer

**DOI:** 10.1038/s41598-017-01066-y

**Published:** 2017-04-19

**Authors:** Yan-jun Lu, Peng Wang, Jing Peng, Xiong Wang, Yao-wu Zhu, Na Shen

**Affiliations:** 1 Department of Laboratory Medicine, Tongji Hospital, Tongji Medical College, Huazhong University of Science and Technology, Wuhan, 430030 China; 2 Institute and Department of Infectious Disease, Tongji Hospital, Tongji Medical College, Huazhong University of Science and Technology, Wuhan, China

## Abstract

Detecting circulating tumour cells (CTCs) is considered as effective and minimally invasive technique to predict the prognosis of patients with metastatic colorectal cancer (CRC), but its clinical validity is still conflicting in patients without metastasis. We performed this meta-analysis to evaluate whether detection of CTCs in the peripheral blood can be used as a prognostic marker for patients with non-metastatic CRC. We performed a comprehensive search of the EMBASE, PubMed, and Web of Science databases (up to September 2016). Meta-analyses were conducted using a random-effects model with the hazard ratio (HR) and 95% confidence interval (95% CI) as the effect measures. Twenty studies including 3,687 patients were eligible for inclusion. Overall analyses demonstrated that the presence of CTCs was significantly associated with aggressive disease progression (HR = 2.57, 95% CI = 1.64–4.02, *P*_*heterogeneity*_ < 0.001, *I* ^*2*^ = 81.0%) and reduced disease survival (HR = 2.41, 95% CI = 1.66–3.51, *P*_*heterogeneity*_ = 0.002, *I* ^*2*^ = 59.7%). Subgroup analyses further supported the prognostic effect of CTCs based on different subsets, including sampling time, detection method and cancer type. Our findings suggest that detection of CTCs in the peripheral blood has the clinical utility to indicate poor prognosis in patients with non-metastatic CRC.

## Introduction

Despite tremendous efforts, colorectal cancer (CRC) remains the third most common cancer, with 1.36 million new cases and 694,000 deaths per year worldwide^1^. Metastasis and recurrence are the primary reasons for the CRC-related deaths. Clinically, approximately 25–50% of patients with early stage CRC develop cancer relapse after radical surgery and adjuvant treatment^2^. In addition, many patients with non-metastatic CRC (especially patients with stage III disease) undergo unnecessary treatment or overtreatment. Therefore, identifying an effective monitoring method to predict prognosis is quite important for CRC management in order to prevent metastasis and recurrence, as well as overtreatment.

According to the “seed and soil theory”, circulating tumour cells (CTCs) released into the peripheral blood from the primary tumour are crucial for the formation of metastases and recurrence^3^. In recent years, accumulating evidence has demonstrated the prognostic role of CTCs in the peripheral blood in several human cancers, such as gastric cancer^4^, head and neck squamous cell carcinoma^5^, prostate cancer^6^, and breast cancer^7^, as well as its subtypes^8^. However, these studies primarily focused on metastatic cancers. Although the prognostic effect of CTCs on CRC has been reported previously^9, 10^, whether CTCs could predict poor outcome in patients with non-metastatic CRC is still inconclusive. Some studies reported that patients with CTCs had a worse prognosis than patients without CTCs^11–19^, while other studies did not support the conclusion^20–27^. Two main detection methods were applied in these studies, namely, reverse transcriptase polymerase chain reaction (RT-PCR) and immunological methods (e.g. CellSearch, Epispot, or CMx platform). For immunological methods, different cut-off values were also used (as shown in Table 1). Moreover, sampling times seem to influence the prognostic effect of CTCs. van Dalum *et al*. surprisingly found that CTCs detected in the weeks after surgery were not significantly associated with CRC progression whereas CTCs detected 2–3 years after surgery were significantly associated with CRC progression^28^. In summary, these discrepancies may result from the small sample size of individual studies, different time points of blood collection, or the use of various detection methods.

**Table 1:** Characteristics of studies included in this meta-analysis.

Study	Country	Patient number	Sampling time	Median follow-up (months)	Disease stage	Cancer type	Detection method	Blood volume (mL)^a^	RT-PCR marker	Detection rate, % (n/N)^b^	Cut-off of CTC-positive	Diagnostic specificity, % (n/N)^c^	Outcomes	HR estimation	Multivariate adjustment
Hardingham^11^	Australia	94	Preoperative	14.8	Dukes’A-C	CRC	RT-PCR	10	CK19, CK20, MUC1, MUC2	20 (19/94)	—	100 (18/18)	OS	Reported	No
		31	Preoperative	14.8	Dukes’s C	CRC	RT-PCR	10	CK19, CK20, MUC1, MUC2	42 (13/31)	—	100 (18/18)	OS	Reported	No
Bessa^20^	Spain	32	Preoperative	42	TNM II	CRC	RT-PCR	20	CEA	38 (12/32)	—	NR	DFS	Extrapolated	No
		27	Preoperative	42	TNM III	CRC	RT-PCR	20	CEA	41 (11/27)	—	NR	DFS	Extrapolated	No
Ito^21^	Japan	99	Postoperative	NR	TNM I-III	CRC	RT-PCR	5–7	CEA	26 (26/99)	—	100 (20/20)	DFS	Extrapolated	No
Bessa^22^	Spain	66	Postoperative	36	TNM I-III	CRC	RT-PCR	20	CEA	55 (36/66)	—	NR	RFS; OS	Extrapolated	No
		24	Postoperative	36	TNM III	CRC	RT-PCR	20	CEA	58 (14/24)	—	NR	RFS	Extrapolated	No
Sadahiro^23^	Japan	99	During surgery	59	TNM I-III	CRC	RT-PCR	NR	CEA	39 (39/99)	—	NR	RFS	Reported	Yes
Douard^24^	France	89	Preoperative	NR	TNM I-III	CRC	RT-PCR	10	CGM2	44 (39/89)	—	NR	RFS	Extrapolated	No
Koch^12^	Germany	82	Postoperative	58	TNM II	CRC	RT-PCR	10	CK20	34 (28/82)	—	100 (98/98)	RFS; CRS	Reported	Yes
Allen-Mersh^13^	UK	113	Postoperative	46.4	Dukes’A-C	CRC	RT-PCR	14	CEA/CK20	30 (34/113)	—	98 (199/203)	RFS	Reported	Yes
Sadahiro^29^	Japan	200	Postoperative	52	TNM I-III	CRC	RT-PCR	NR	CEA	22 (44/200)	—	NR	DFS; OS	Reported	Yes
Koyanagi^14^	USA	34	Preoperative	34	TNM I-III	CRC	RT-PCR	9	c-MET, MAGE-A3, GalNAc-T, CK20	47 (16/34)	—	100 (47/47)	OS	Reported	Yes
Uen^15^	China	438	Preoperative and postoperative	44	TNM I-III	CRC	RT-PCR	4	hTERT, CK19, CK20, CEA	31 (137/438)	—	NR	RFS	Reported	Yes
		287	Preoperative and postoperative	44	TNM I-III	Colon cancer	RT-PCR	4	hTERT, CK19, CK20, CEA	32 (92/287)	—	NR	RFS	Extrapolated	No
		151	Preoperative and postoperative	44	TNM I-III	Rectal cancer	RT-PCR	4	hTERT, CK19, CK20, CEA	30 (45/151)	—	NR	RFS	Extrapolated	No
Iinuma (training)^16^	Japan	420	Preoperative	NR	Dukes’B-C	CRC	RT-PCR	10	CEA, CK19, CK20, CD133	25 (106/420)	—	NR	DFS; OS	Reported	Yes
		176	Preoperative	NR	Dukes’B	CRC	RT-PCR	10	CEA, CK19, CK20, CD133	23 (41/176)	—	NR	DFS; OS	Reported	Yes
		150	Preoperative	NR	Dukes’C	CRC	RT-PCR	10	CEA, CK19, CK20, CD133	38 (57/150)	—	NR	DFS; OS	Reported	Yes
		268	Preoperative	NR	Dukes’B-C	Colon cancer	RT-PCR	10	CEA, CK19, CK20, CD133	26 (69/268)	—	NR	DFS; OS	Extrapolated	No
		152	Preoperative	NR	Dukes’B-C	Rectal cancer	RT-PCR	10	CEA, CK19, CK20, CD133	24 (37/152)	—	NR	DFS; OS	Extrapolated	No
Iinuma (validation)^16^	Japan	315	Preoperative	NR	Dukes’B-C	CRC	RT-PCR	10	CEA, CK19, CK20, CD133	24 (75/315)	—	NR	DFS; OS	Reported	Yes
		143	Preoperative	NR	Dukes’B	CRC	RT-PCR	10	CEA, CK19, CK20, CD133	22 (32/143)	—	NR	DFS; OS	Reported	Yes
		97	Preoperative	NR	Dukes’C	CRC	RT-PCR	10	CEA, CK19, CK20, CD133	36 (35/97)	—	NR	DFS; OS	Reported	Yes
		203	Preoperative	NR	Dukes’B-C	Colon cancer	RT-PCR	10	CEA, CK19, CK20, CD133	23 (46/203)	—	NR	DFS; OS	Extrapolated	No
		112	Preoperative	NR	Dukes’B-C	Rectal cancer	RT-PCR	10	CEA, CK19, CK20, CD133	25 (28/112)	—	NR	DFS; OS	Extrapolated	No
Lu^17^	China	141	Postoperative	40	TNM II-III	Colon cancer	RT-PCR	4	hTERT, CK19, CK20, CEA	36 (51/141)	—	NR	RFS; OS	Reported for RFS; Extrapolated for OS	Yes for RFS; No for OS
Deneve ^25^	France	60	Preoperative	36	M0	CRC	Epispot	10–20	CK19	12 (7/60)	27 CTCs/10–20 mL	100 (20/20)	CRS	Extrapolated	No
Lu^30^ ^*d*^	China	90	Postoperative	36	TNM III	Colon cancer	RT-PCR	4	hTERT, CK19, CK20, CEA	23 (21/90)	—	NR	DFS; OS	Reported	Yes
Bork^18^	Germany	239	Preoperative	28	TNM I-III	CRC	CellSearch	7.5	—	8.8 (21/239)	1 CTC/7.5 mL	NR	PFS; OS	Reported	Yes
Sotelo^26^	Spain	472	Postoperative	40	TNM III	CRC	CellSearch	7.5	—	35 (166/472)	1 CTC/7.5 mL	NR	DFS; OS	Reported	Yes
van Dalum^28^	Netherlands	183	Preoperative	61	TNM I-III	CRC	CellSearch	7.5	—	24 (44/183)	1 CTC/30 mL	NR	RFS; CCRD	Reported	Yes
		146	Postoperative	61	TNM I-III	CRC	CellSearch	7.5	—	20 (29/146)	1 CTC/30 mL	NR	CCRD	Extrapolated	No
Kust^27^	Croatia	82	Preoperative	50	TNM I-III	CRC	RT-PCR	10	CK20	73 (60/82)	—	70 (16/23)	RFS; OS	Extrapolated	No
		82	Postoperative	50	TNM I-III	CRC	RT-PCR	10	CK20	74 (61/82)	—	70 (16/23)	RFS; OS	Extrapolated	No
Tsai^19^	China	84	Preoperative	NR	TNM I-III	CRC	CMx platform	2	CK20	43 (36/84)	5 CTCs/2 mL	100 (27/27)	DFS	Reported	Yes

Abbreviations: NR, not reported; M0, non-metastasis; M1, metastasis; RT-PCR, reverse transcriptase polymerase chain reaction; OS, overall survival; DFS, disease-free survival; RFS, recurrence-free survival; CRS, cancer-related survival; CCRD, colon cancer related death.

^a^It referred to the sample blood volume used for CTC isolation in each study.

^b^It referred to the number of CTC-positive patients (n) per total number of patients (N) included in each study.

^c^It referred to the number of CTC-negative subjects (n) per total number of healthy controls (N) included in each study.

^d^The study of Lu (2013) was removed in the overall analysis because it had overlapping cases with the study of Lu (2011), but it was included in the subgroup analysis based on cancer type.

Therefore, we performed this meta-analysis to comprehensively and quantitatively evaluate the prognostic significance of CTCs detected in the peripheral blood of patients with non-metastatic CRC. Moreover, we investigated the potential role of CTCs in different subgroups based on patient number, sampling time, detection method, detection rate, disease stage, or cancer type. The outcomes of interest were disease progression (including disease-free survival [DFS] and recurrence-free survival [RFS]) and disease survival (including overall survival [OS], cancer-related survival [CRS] and colon cancer related death [CCRD]).

## Results

### Characteristics of the included studies

**Figure 1:**
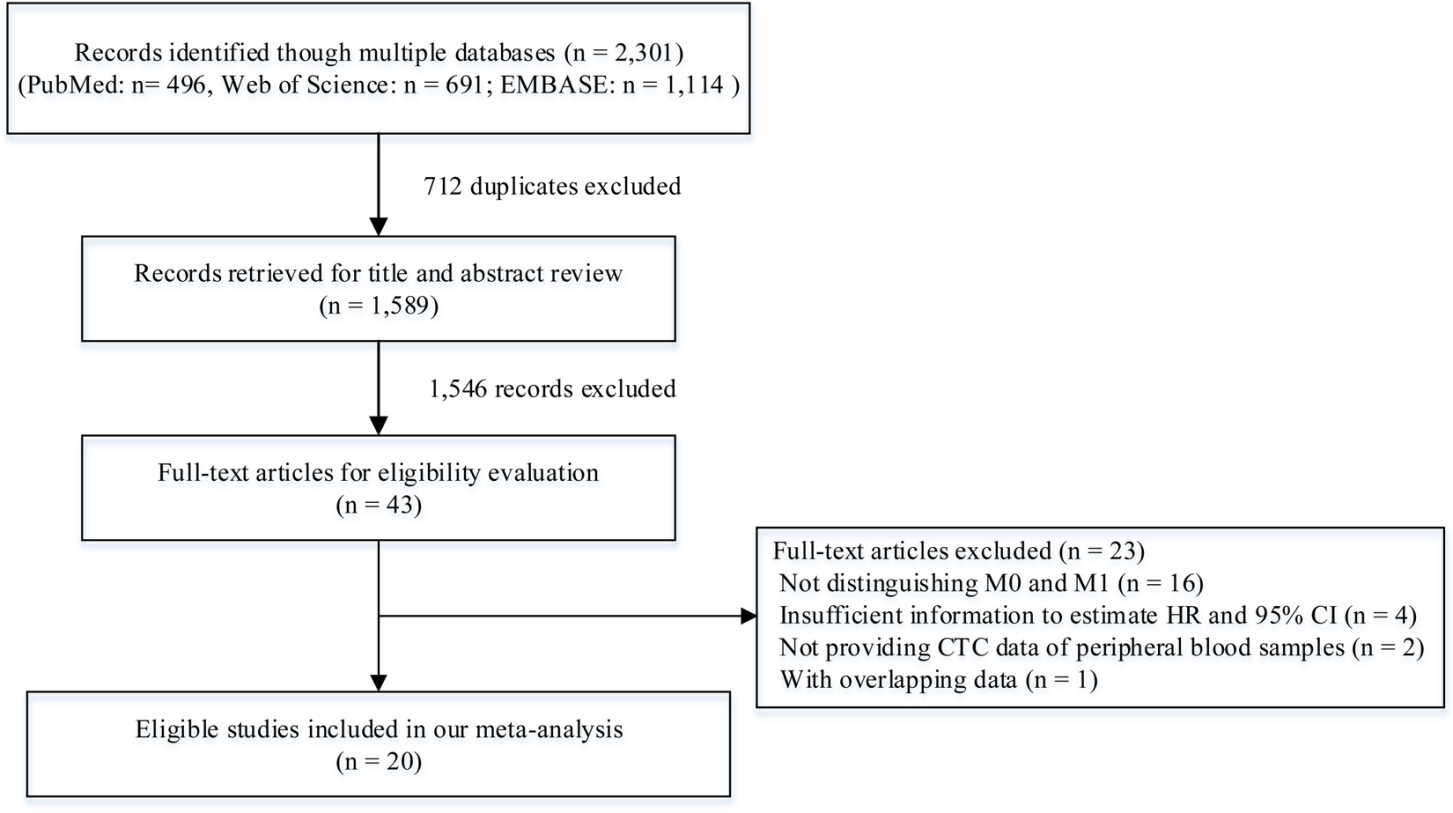
A flowchart of literature search.

As shown in Fig. 1, a total of 2,301 records were initially identified from the EMBASE, PubMed, and Web of Science databases. By screening the title and abstract, we excluded 712 duplicates and 1,546 unrelated records, and then retrieved 43 relevant full-text articles. Twenty-three studies were further removed because of failure to distinguish data of early stage (M0) and metastatic stage (M1) CRC (n = 16), insufficient information to estimate the insufficient information to estimate the hazard ratio (HR) and 95% confidence interval (95% CI) (n = 4), failure to report CTC data of peripheral blood samples (n = 2), or having populations overlapping with another study (n = 1). Finally, 20 eligible studies were included in this meta-analysis^11–30^.

The 20 studies including 3,687 patients with non- metastatic CRC were performed in Asia, Europe, North America and Oceania. Eight studies evaluated the effect of CTCs at a “preoperative” time point^11, 14, 16, 18–20, 24, 25^, eight studies evaluated at a “postoperative” time point^12, 13, 17, 21, 22, 26, 29, 30^, two studies evaluated at “preoperative” and “postoperative” time points^27, 28^, one study evaluated at “preoperative and postoperative” (persistent CTC-positive before and after surgery)^15^, and one study evaluated at a “during surgery” time point^23^. CTCs in these studies were detected by two types of detection methods, RT-PCR and immunological methods (e.g. CellSearch, Epispot, or CMx platform). The detection rates ranged from 8.8% to 74%. Table 1 summarises the characteristics of the 20 included studies. Eighty percent (16/20) of these studies were high-quality (quality score ≥6) according to the Newcastle-Ottawa scale (Supplementary Table S1).

### Overall analyses

**Figure 2:**
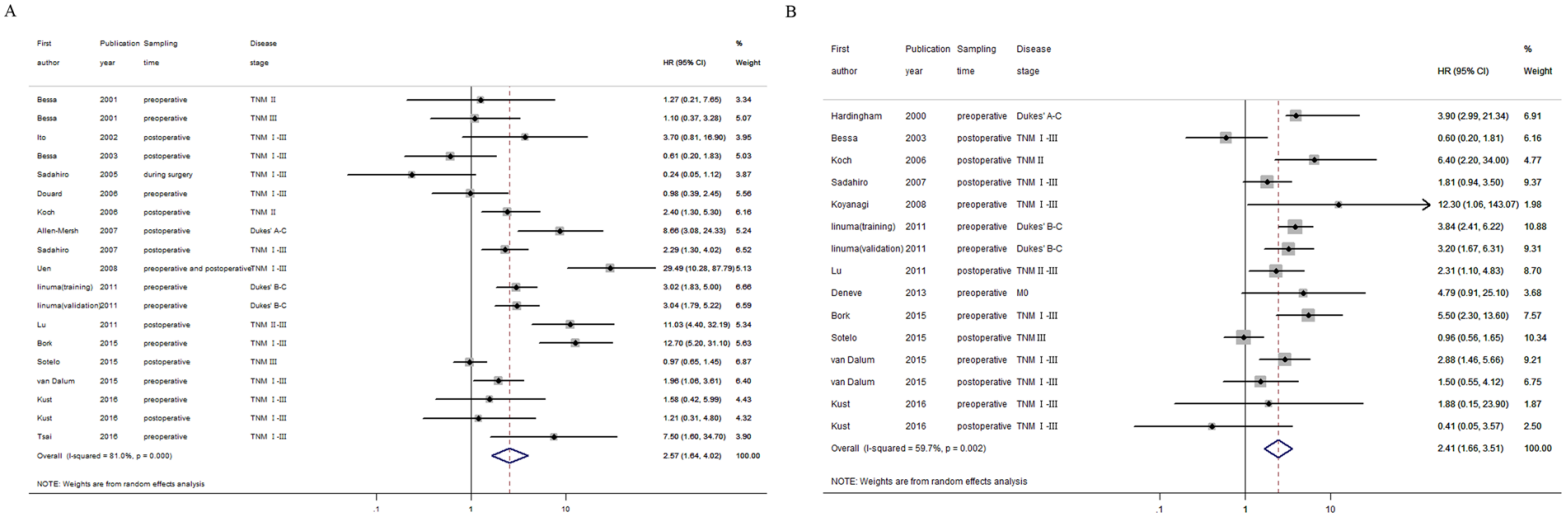
Overall forest plots of the prognostic effect of CTCs detected in the peripheral blood on the disease progression (**A**) or survival (**B**) in patients with non-metastatic CRC.

HRs for disease progression (DFS and RFS) were provided by 16 studies^12, 13, 15–24, 26–29^ including 3,263 patients with non-metastatic CRC. In three studies, more than one HR was collected from each trial by using different disease stages^20^, research populations (e.g. training set and validation set)^16^, or sampling time points^27^. The overall analysis demonstrated that patients who were CTC-positive with non-metastatic CRC had a significant higher risk of disease progression (HR = 2.57, 95% CI = 1.64–4.02, *P*_*heterogeneity*_ < 0.001, *I* ^*2*^ = 81.0%; Fig. 2A).

HRs for disease survival (OS, CRS, CCRD) were provided by 12 studies^11, 12, 14, 16–18, 22, 25–29^ including 2,616 patients with non-metastatic CRC. More than one HR was collected in three studies^16, 27, 28^ because of the same reasons as mentioned above. The overall analysis showed that, compared with patients who were CTC-negative with non-metastatic CRC, patients who were CTC-positive had a two-fold increased risk of worse survival (HR = 2.41, 95% CI = 1.66–3.51, *P*_*heterogeneity*_ = 0.002, *I* ^*2*^ = 59.7%; Fig. 2B).

### Meta-regression and subgroup analyses

Because of the significant heterogeneity among studies, we conducted a meta-regression analysis to investigate potential sources (Table 2). Results showed that patient number (*P* = 0.023) and detection rate (*P* = 0.022) were significant factors affecting heterogeneity for disease progression. Meta-regression analysis also indicated that sampling time (*P* = 0.001) and detection rate (*P* = 0.011) were responsible for heterogeneity for disease survival.

**Table 2:** Univariate meta-regression analyses for exploring potential sources of heterogeneity.

Factors^a^	Disease progression			Disease survival		
	Coefficient	SE	*P*	Coefficient	SE	*P*
Publication year	0.055	0.052	0.309	−0.003	0.047	0.944
Patient number	1.154	0.461	**0**.**023**	0.095	0.469	0.843
Sampling time	−0.068	0.452	0.882	−0.980	0.233	**0**.**001**
Detection method	0.330	0.646	0.617	−0.055	0.415	0.897
Median follow-up	−0.214	1.024	0.838	−0.517	0.490	0.313
Detection rate	−1.154	0.458	**0**.**022**	−0.913	0.308	**0**.**011**
HR estimation	−1.069	0.515	0.053	−0.655	0.412	0.136
Multivariate adjustment	−1.069	0.515	0.053	0.538	0.403	0.205

Abbreviations: SE, standard error of the coefficient.

^a^Patient number referred to <100 versus ≥100. Sampling time referred to preoperative versus postoperative. Detection method referred to RT-PCR versus immunological methods. Median follow-up referred to <40 months versus ≥40 months. Detection rate referred to <35% versus ≥35% (35% was the median of the detection rate of each study included in overall analysis). HR estimation referred to reported in articles versus extrapolated by data. Multivariate adjustment referred to yes versus no.

Moreover, we performed subgroup analyses to further assess the prognostic value of CTCs in different subsets (Table 3). In the subgroup analysis based on patient number (≥100 or not), a significant prognostic effect of CTC detection was only identified in the analysis of studies with ≥100 cases (Disease progression: HR = 4.40, 95% CI = 2.32–8.35, *P*_*heterogeneity*_ < 0.001, *I* ^*2*^ = 88.6%; Disease survival: HR = 2.40, 95% CI = 1.59–3.62, *P*_*heterogeneity*_ = 0.004, *I* ^*2*^ = 66.2%). Subgroup analyses based on sampling time confirmed that whether performed before surgery or after, detection of CTCs in the peripheral blood could predict worse disease progression (Preoperative: HR = 2.57, 95% CI = 1.57–4.21, *P*_*heterogeneity*_ = 0.003, *I* ^*2*^ = 65.5%; Postoperative: HR = 2.41, 95% CI = 1.24–4.69, *P*_*heterogeneity*_ < 0.001, *I* ^*2*^ = 81.1%). A prognostic role of CTCs in disease survival was also shown in the analysis of studies collecting blood samples at the preoperative point (HR = 3.71, 95% CI = 2.78–4.96, *P*_*heterogeneity*_ = 0.903, *I* ^*2*^ = 0.0%), as well as at the postoperative point (HR = 1.47, 95% CI = 0.89–2.42, *P*_*heterogeneity*_ = 0.051, *I* ^*2*^ = 52.0%), although the latter did not reach statistical significance. Subgroup analyses based on detection rate showed that a CTC-positive detection rate ≥35% tended to indicate an unfavourable prognosis (Disease progression: HR = 1.41, 95% CI = 0.75–2.66, *P*_*heterogeneity*_ < 0.001, *I* ^*2*^ = 71.4%; Disease survival: HR = 1.28, 95% CI = 0.65–2.51, *P*_*heterogeneity*_ = 0.083, *I* ^*2*^ = 48.6%), although statistical significance was not reached. Similar results were also observed in the subgroup analysis of TNM stage III disease (Disease progression: HR = 1.55, 95% CI = 0.55–4.39, *P*_*heterogeneity*_ = 0.004, *I* ^*2*^ = 77.6%; Disease survival: HR = 2.06, 95% CI = 0.38–10.99, *P*_*heterogeneity*_ = 0.014, *I* ^*2*^ = 83.4%). In other subgroup analyses, detection of CTCs showed a prognostic value for both disease progression and survival for non-metastatic CRC, under various conditions including different detection methods (RT-PCR or immunological methods), different disease stages (Dukes’ B or Dukes’ C), different cancer types (colon cancer or rectal cancer), or after multivariate adjustment (shown in Table 3).

**Table 3:** Subgroup analyses of the prognostic effect of CTCs detected in the peripheral blood.

	Disease progression				Disease survival			
	n	HR (95% CI)	*P*_*heterogeneity*_	*I* ^*2*^ (%)	n	HR (95% CI)	*P*_*heterogeneity*_	*I* ^*2*^ (%)
Patient number								
<100	10	1.37 (0.82–2.31)	0.060	45.0	7	2.47 (0.99–6.16)	0.029	57.3
≥100	9	4.40 (2.32–8.35)	<0.001	88.6	8	2.40 (1.59–3.62)	0.004	66.2
Sampling time								
Preoperative	9	2.57 (1.57–4.21)	0.003	65.5	8	3.71 (2.78–4.96)	0.903	0.0
Postoperative	8	2.41 (1.24–4.69)	<0.001	81.1	7	1.47 (0.89–2.42)	0.051	52.0
Detection method								
RT-PCR	15	2.43 (1.49–3.96)	<0.001	76.2	10	2.52 (1.64–3.90)	0.038	49.2
Immunological methods^a^	4	3.32 (1.04–10.61)	<0.001	90.2	5	2.33 (1.13–4.82)	0.006	72.4
Detection rate (%)								
<35	9	4.29 (2.61–7.08)	<0.001	76.0	9	3.18 (2.43–4.15)	0.385	6.1
≥35	10	1.41 (0.75–2.66)	<0.001	71.4	6	1.28 (0.65–2.51)	0.083	48.6
TNM stage								
TNM II	2	2.21 (1.15–4.24)	0.518	0.0	1	6.40 (1.63–25.16)	—	—
TNM III	4	1.55 (0.55–4.39)	0.004	77.6	2	2.06 (0.38–10.99)	0.014	83.4
Dukes’ stage								
Dukes’ B	2	3.25 (1.89–5.57)	0.890	0.0	2	3.38 (1.73–6.61)	0.829	0.0
Dukes’ C	2	3.13 (1.93–5.09)	0.853	0.0	3	2.63 (1.67–4.12)	0.981	0.0
Cancer type								
Colon cancer	4	5.62 (3.82–8.28)	0.519	0.0	3	2.89 (1.82–4.59)	0.607	0.0
Rectal cancer	3	3.88 (2.01–7.48)	0.804	0.0	2	4.43 (1.43–13.68)	0.474	0.0
Multivariate adjustment								
Yes	12	3.62 (2.06–6.37)	<0.001	86.4	8	2.90 (1.78–4.72)	0.002	69.9
No	7	1.15 (0.72–1.83)	0.691	0.0	7	1.78 (0.98–3.24)	0.128	39.5

^a^Immunological methods included CellSearch, Epispot and CMx platform. CellSearch is to use anti-EpCAM antibody coated on magnetic beads for cell capture and then identify (CK)8/18/19+/DAPI+/CD45− cells as CTCs by immunostaining. CMx platform is also a EpCAM-dependent method to capture CK20+/DAPI+/CD45− cells as CTCs in peripheral blood. Epispot is an EpCAM-independent method based on capturing CK19− releasing cells after a depletion of hematopoietic CD45+ cells.

### Sensitivity analyses and publication bias

**Figure 3:**
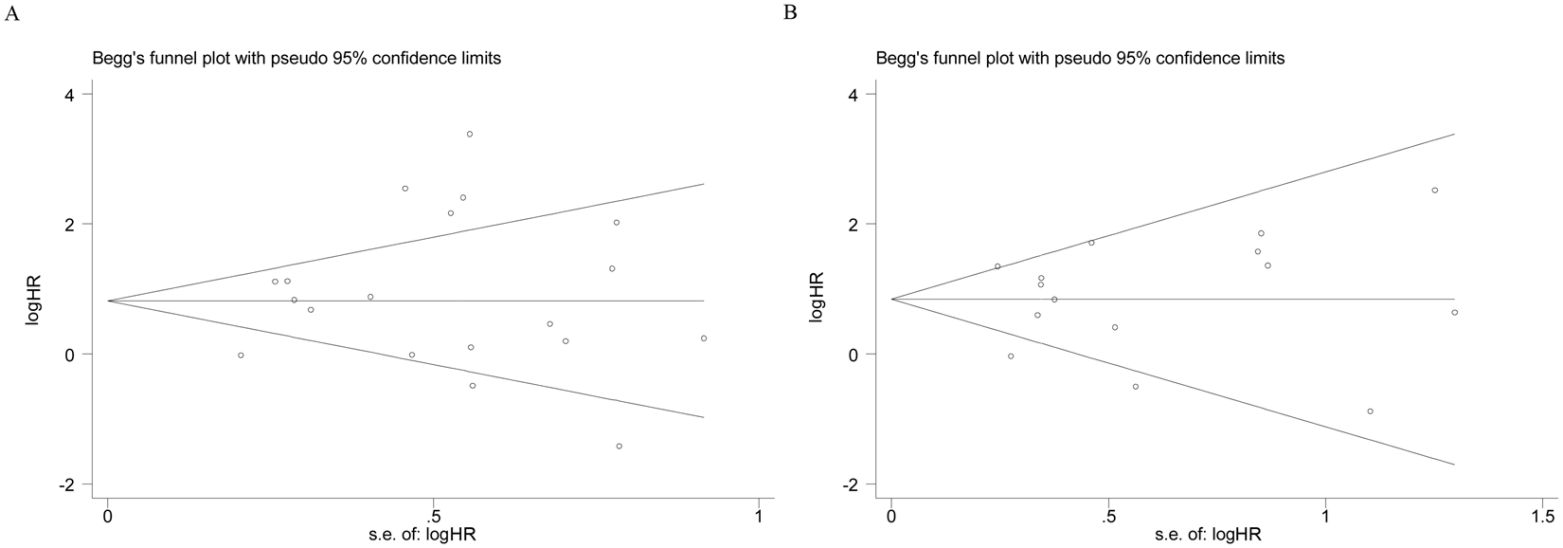
Funnel plots of the prognostic effect of CTCs detected in the peripheral blood on the disease progression (**A**) or survival (**B**) in patients with non-metastatic CRC.

Sensitivity analyses indicated that our pooled results were quite stable for both disease progression (Supplementary Figure S1) and disease survival (Supplementary Figure S2). Moreover, the funnel plots and the Begg’s and Egger’s tests showed no evidence of publication bias on the pooled analysis of disease progression (Fig. 3A; *P*_*Egger’s test*_ = 0.413, *P*_*Begg’s test*_ = 0.624) and disease survival (Fig. 3B; *P*_*Egger’s test*_ = 0.830, *P*_*Begg’s test*_ = 1.000).

## Discussion

The prognostic significance of CTCs has been confirmed in several metastatic cancers, but rarely evaluated in early-stage cancers. This meta-analysis comprehensively summarised the relevant studies and provided strong evidence that the presence of CTCs in the peripheral blood could predict a poor disease progression and survival in patients with non-metastatic CRC.

In 2010, Rahbari *et al*. performed an excellent meta-analysis that demonstrated the unfavourable prognostic role of CTCs in patients with primary CRC^9^. However, in their subgroup analysis to evaluate CTCs detected in peripheral/central blood from patients with CRC stage I-III, only one study was included for evaluation of RFS (*I* ^*2*^ = 78%) and five studies were included for evaluation of OS (*I* ^*2*^ = 69%). It is difficult to obtain a convincing conclusion based on the limited study number and high heterogeneity. In 2015, Huang *et al*. conducted an updated meta-analysis to evaluate the prognostic utility of CTCs detected in the peripheral blood by the CellSearch System^10^. However, their work also did not distinguish stage M0 from M1. Our study addressed these issues and further confirmed the potential clinical utility of CTC detection in patients with localised cancers. Furthermore, we performed subgroup analyses to thoroughly assess the prognostic effect of CTCs based on patient number, sampling time, detection method, detection rate, disease stage and cancer type.

When the studies were divided into two groups based on patient number, the pooled results of the group with lower patient numbers (n < 100) failed to reach statistical significance in terms of both disease progression and survival (Table 3). Patient number was also identified as a potential source of the heterogeneity by meta-regression analyses. This might explain, at least partially, why non-significant results in this research field were often observed in these “small” studies. Preoperative CTCs and postoperative CTCs in the peripheral blood usually indicate different clinical events, respectively. The presence of CTCs before surgery reflect the invasion of the primary tumour into the blood and could determine those subpopulations at high risk for recurrence; the presence of CTCs after surgery could be considered as an early indicator of the undetectable metastasis^19, 28^. Our results showed a significant association between preoperative CTCs and poor disease progression and survival, suggesting that detecting CTCs before surgery is a promising method to distinguish patients with high-risk CRC at early stages. A significant association was also identified in the analysis of disease progression in the postoperative subgroup, suggesting the prognostic value of postoperative CTCs in patients with non-metastatic CRC. However, in the analysis of disease survival, postoperative CTCs did not significantly indicate a poor survival. A possible explanation was that there were other factors influencing the survival of patients (e.g. death due to accidents or other diseases), and our sample size was not large enough to identify the significance. We also performed subgroup analysis based on detection rate, which was the median of the detection rate of each study included in the overall analysis. We noticed inconsistent results, in that the subset with a detection rate <35% showed a significant association while the subset with detection rate ≥35% only showed a similar trend but failed to reach statistical significance. When we performed subgroup analyses based on disease stage, we observed consistently significant associations without heterogeneity at most disease stages, except for TNM stage III. This included a limited number of studies (n < 5); significant heterogeneity (*I* ^*2*^ = 77.6% for disease progression and *I* ^*2*^ = 83.4% for disease survival) might be responsible for the conflicting results of TNM stage III disease. In addition, other subgroup analyses suggested that CTCs had a significantly prognostic effect in patients with non-metastatic CRC regardless of detection method, cancer type, or multivariate adjustment.

Several limitations should be addressed as follows. Firstly, because of the limited number of studies focusing on separate disease stages, we could not fully evaluate the prognostic value of CTCs in patients with CRC at each clinical stage, especially TNM I or Dukes’ stage A. Secondly, some of the included studies did not provide multivariate adjusted HRs; in this case, we recorded unadjusted HRs or extrapolated them by reported data instead. Thus, our pooled results carry a risk of bias due to potential confounders in the original studies. However, subgroup analysis based on multivariate adjustment showed a significant result for studies with adjusted HRs and a similar trend of results for studies with unadjusted HRs. This suggests that suggested that potential confounders in the original studies might not affect the conclusions of our meta-analysis. Thirdly, significant heterogeneity among studies was observed in the overall analyses. Although meta-regression analyses identified patient number, sampling time, and detection rate as significant heterogeneous factors, subgroup analyses based on these factors also showed obvious heterogeneity, which suggests that there were other potentially influencing factors. Finally, the influence of adjuvant therapies on the prognostic effect of CTCs was not evaluated in our work since few included studies provided such data. In spite of these limitations, our work is the first meta-analysis to assess the prognostic utility of CTCs detected in the peripheral blood for patients with non-metastatic CRC.

In conclusion, our meta-analysis strongly suggests that the detection of CTCs in peripheral blood is a clinically promising predictor of worse disease progression and survival for patients with non-metastatic CRC. More high-quality cohort studies with refined designs are still required to further validate our results.

## Methods

### Literature search and eligibility criteria

Our meta-analysis was conducted according to the statement of the Preferred Reporting Items for Systematic Reviews and Meta-Analyses (PRISMA)^31^. We performed a comprehensive electronic search in multiple databases including EMBASE, PubMed and Web of Science through September 2016, without any restriction. The search items were combinations of “circulating tumour cells”, “micrometastasis”, “disseminated tumour cells”, “isolated tumour cells”, “occult tumour cells”, “colorectal”, “colon”, “rectal”, “cancer”, “tumour”, “neoplasm”, “malignancy”, “carcinoma”, “prognosis”, “survival” and “recurrence”. We also carefully reviewed the reference lists of the identified articles to retrieve potentially relevant studies. Only articles in English published on peer-reviewed journals were included.

Eligible studies were included if they met the following criteria: (1) cohort studies evaluating the prognostic significance of CTCs detected in patients with non-metastatic CRC; (2) studies reporting HRs and 95% CIs, or providing sufficient data to extrapolate these outcome measures; (3) samples collected from peripheral blood. The exclusion criteria were as follows: (1) studies not distinguishing stage M0 and M1; (2) outcome measures not reporting or impossible to be calculated from originally published data; (3) studies with overlapping data or patients. If a study had overlapping data with other studies, we kept the study with larger sample size. Two independent authors performed the literature search and study selection. Discrepancies were resolved by consensus or consultation of a third party.

### Data extraction and quality evaluation

The following items were independently extracted by two authors from each eligible study: first author, publication year, country, patient number, sampling time, the median follow-up, disease stage, cancer type, detection method, detection rate, outcomes, HRs and 95% CIs, and so on. If more than one peripheral blood sample per patient was collected at different points in time, each sampling time point was recorded, and all these results were considered as independent data sets. We used the Newcastle-Ottawa (NOS) scale (Supplemental Table S1)^32^ to evaluate the quality of each included study. The NOS score ranges from “0” to “9” and a score ≥6 indicates high quality. Discrepancies were resolved through consensus.

### Statistical analysis

Multivariate adjusted HR and 95% CI was preferentially chosen from each eligible study, if available. For those studies not reporting HR and 95% CI, we extrapolated the values using the methods of Parmar^33^ and Tierney^34^. A random-effects model was performed to pool these HRs and 95% CIs. Heterogeneity was examined by Cochran’s Q test and further quantified by the *I* ^*2*^ index. *P* < 0.10 or *I* ^*2*^ > 50% suggest significant heterogeneity among the included studies^35^. We also conducted meta-regression analysis to explore the possible sources of heterogeneity. To further investigate the effect of CTCs on the prognosis of non-metastatic CRC, we carried out subgroup analyses based on potential modifiers including patient number, sampling time, detection method, detection rate, TNM stage, Dukes’ stage, and cancer type. In addition, we assessed the stability of the pooled results by one-way sensitivity analysis and examined the publication bias by Egger’s^36^ and Begg’s^37^ tests. All statistical tests were conducted using Stata 12.1 software (College Station, TX, USA). A two-sided P ≤ 0.05 was considered as significant, unless otherwise specified.

## Electronic supplementary material

Supplemental tables and figures
