# Supplementary material for: Meta-analysis Reveals the Prognostic Value of Circulating Tumour Cells Detected in the Peripheral Blood in Patients with Non-Metastatic Colorectal Cancer
Source: Sci Rep. 2017 Apr 19;7:905. doi: 10.1038/s41598-017-01066-y (PMC5430457; doi:10.1038/s41598-017-01066-y)

Meta-analysis Reveals the Prognostic Value of Circulating Tumour Cells Detected in the Peripheral Blood in Patients with Non-Metastatic Colorectal Cancer

Yan-jun Lu [1*], Peng Wang [2*], Jing Peng [1], Xiong Wang [1], Yao-wu Zhu [1], Na Shen [1]

[1] Department of Laboratory Medicine, Tongji Hospital, Tongji Medical College, Huazhong University of Science and Technology, Wuhan 430030, China.

[2] Institute and Department of Infectious Disease, Tongji Hospital, Tongji Medical College, Huazhong University of Science and Technology, Wuhan, China.

Correspondence to:

Na Shen, Department of Laboratory Medicine, Tongji Hospital, Tongji Medical College, Huazhong University of Science and Technology, Wuhan 430030, China. Tel. : +86-27-83663414, E-mail: shenna@tjh.tjmu.edu.cn

[*] These authors contributed equally to this work.

Supplementary Table S1. Quality assessment of included studies by Newcastle-Ottawa scale

Study	Selection (0-4)				Comparability (0-2)		Outcome (0-3)			Score
	REC	SNEC	AE	DO	SC	AF	AO	FU	AFU	
Hardingham (2000) [11]		1	1	1			1	1		5
Bessa (2001) [20]		1	1	1			1	1	1	6
Ito (2002) [21]		1	1	1			1	1		5
Bessa (2003) [22]		1	1	1	1		1	1	1	7
Sadahiro (2005) [23]		1	1	1		1	1	1	1	7
Douard (2006) [24]		1	1	1			1	1	1	6
Koch (2006) [12]		1	1	1		1	1	1	1	7
Allen-Mersh (2007) [13]		1	1	1		1	1	1		6
Sadahiro (2007) [29]		1	1	1		1	1	1	1	7
Koyanagi (2008) [14]		1	1	1		1	1	1		6
Uen (2008) [15]		1	1	1		1	1	1	1	7
Iinuma (2011) [16]		1	1	1		1	1	1		6
Lu (2011) [17]		1	1	1		1	1	1	1	7
Deneve (2013) [25]		1	1	1			1	1		5
Lu (2013) [30]		1	1	1		1	1	1	1	7
Bork (2015) [18]		1	1	1		1	1	1		6
Sotelo (2015) [26]	1	1	1	1		1	1	1		7
van Dalum (2015) [28]		1	1	1		1	1	1		6
Kust (2016) [27]		1	1	1			1	1		5
Tsai (2016) [19]		1	1	1		1	1	1		6

Abbreviations: REC, representativeness of the exposed cohort; SNEC, selection of the non-exposed cohort; AE, ascertainment of exposure; DO, demonstration that outcome of interest was not present at start of study; SC, study controls for sex and age; AF, study controls for any additional factor (eg. disease stage, adjuvant treatment); AO, assessment of outcome; FU, follow-up long enough (36 months) for outcomes to occur; AFU, adequacy of follow up of cohorts.

Supplemental Figure S1. Sensitivity analyses of the prognostic effect of CTCs detected in the peripheral blood on the disease progression in non-metastatic CRC patients.

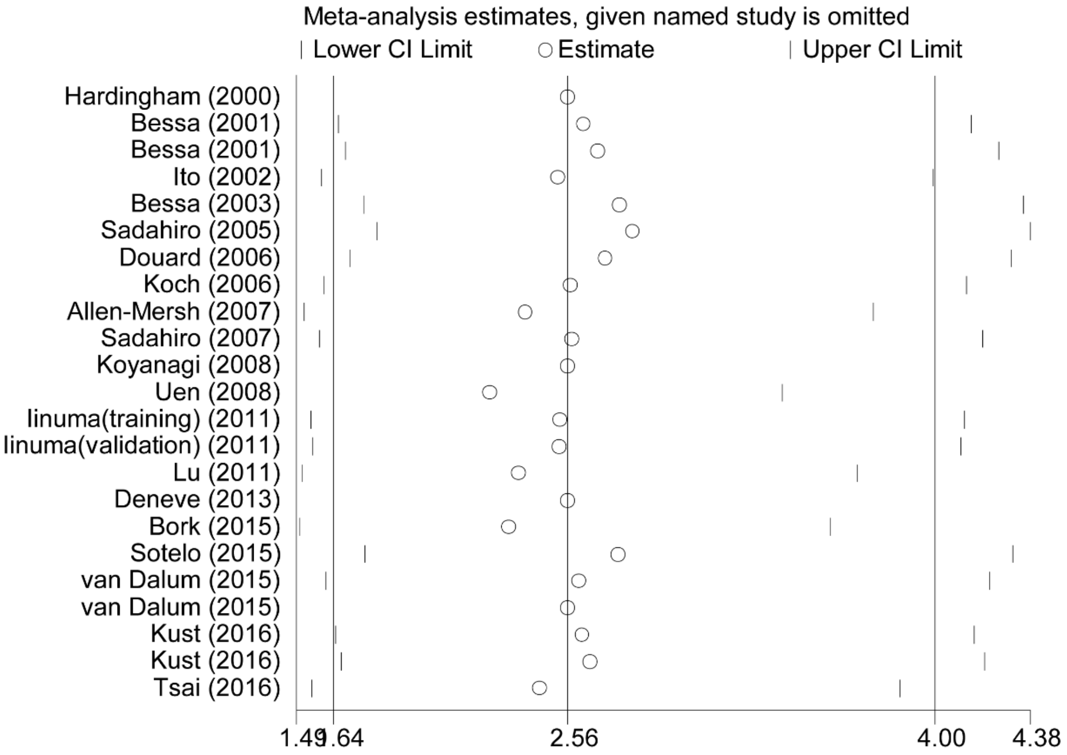

Supplemental Figure S2. Sensitivity analyses of the prognostic effect of CTCs detected in the peripheral blood on the disease survival in non-metastatic CRC patients.

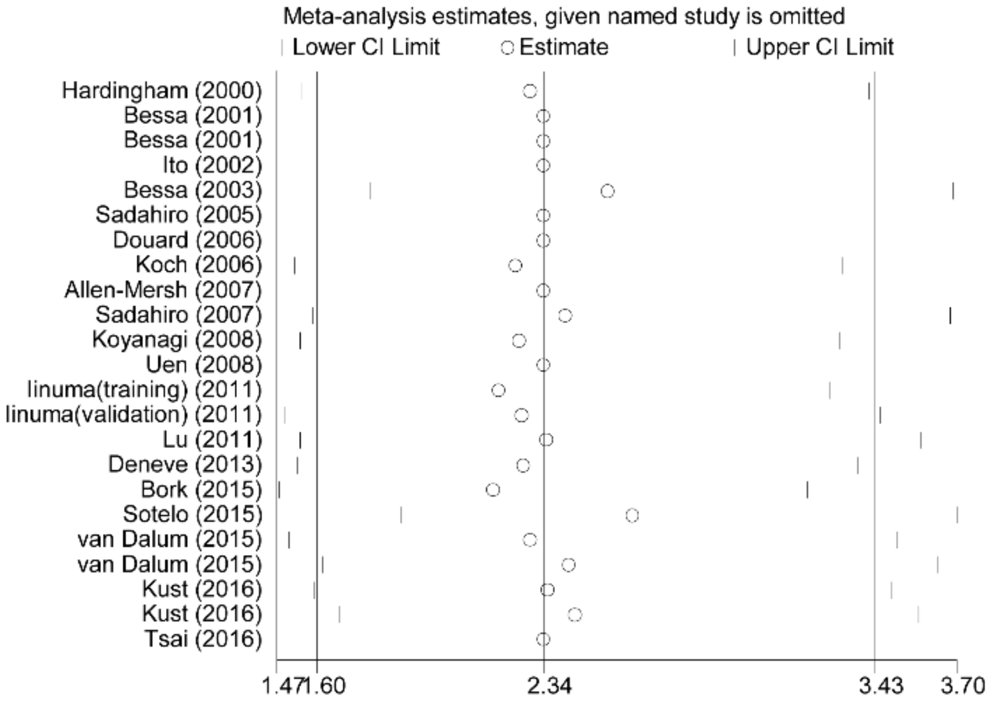

Supplement: Supplementary file 1 — Supplemental tables and figures [file 41598_2017_1066_MOESM1_ESM.pdf]
